# Long-Term Impact of War, Civil War, and Persecution in Civilian Populations—Conflict and Post-Traumatic Stress in African Communities

**DOI:** 10.3389/fpsyt.2020.00020

**Published:** 2020-02-25

**Authors:** Seggane Musisi, Eugene Kinyanda

**Affiliations:** ^1^ Department of Psychiatry, College of Health Sciences, Makerere University, Kampala, Uganda; ^2^ College of Health Sciences, Makerere University, Kampala, Uganda

**Keywords:** war, conflict, trauma, post-traumatic stress disorder (PTSD), oppression, mental illness, Africa

## Abstract

This chapter describes how chronic conflict, warfare, and persecution, as lived experiences, have created significant mental distress in communities on the African continent. There is a growing body of research that highlights increasing mental distress in Africa e.g., about sexuality, health, disease, modernity, climate, politics, culture, religion, ethnicities, race, economies etc. Many of these stresses and uncertainties are driven by political persecution, war, and conflict. This has shaped many African people’s attitudes and government policies and an increasing scholarly interest in exploring these “uncertainties and mental distresses in Africa.” The chapter will show how trauma, as seen in conflict/post-conflict settings in Africa, causes significant mental stress and associated social problems as well as medically-defined PTSD syndromes, anxiety, and depression which cause much morbidity and retard development in many African communities. Taking a classical look at post-traumatic stress disorder, PTSD, the chapter explores the presentation of the various physical and mental clinical syndromes related to war-trauma on the African continent and the consequent health-seeking behaviors of the African peoples in this regard. The term “culture-bound PTSD syndromes” will be introduced and discussed in the broader context of treatment, rehabilitation, and prevention on the continent and worldwide. It will also discuss the dilemma of the vicious cycles of trauma driven by appetitive aggression in today’s Africa which portends to further retard socio-economic development and drives the trans-generational perpetuation of ethnic-based conflicts including genocides. Despite this mass traumatization, the chapter points to the virtual absence of post-conflict mental health policies in almost all African countries, hence leading to discussions of “best-practices” recommendations.

## Introduction

There is a growing body of research that highlights increasing social and psychological distress in Africa (1). This is most often experienced around such areas as conflict, war, sexuality, health, disease, conflicted modernity, climate, politics, culture, religion, ethnicities, race, poverty, famine, governance, economies, migrations etc. (2). In more recent years, many of these psycho-social problems have been driven by war-conflict (3). These have shaped the current African peoples’ attitudes, politics, government policies, and population movements and migrations. They have thus attracted increasing scholarly interest in exploring these African “problems.” This chapter will take a classical look at post-traumatic stress disorder, PTSD, to explore the presentation of the various physical and mental clinical syndromes related to war-trauma on the African continent throughout the ages and the consequent health-seeking behaviors of the African peoples in this regard.

The chapter describes how chronic warfare, as a lived experience, creates significant social and psychological distress in and about the African continent. It will show how trauma, as seen in conflict/post-conflict settings in Africa, causes not only significant psycho-social problems but also medically well-defined psychiatric syndromes such as PTSD, anxiety, and depression which cause much morbidity and retard development in many African communities. The chapter will also discuss the dilemma of the vicious cycles of trauma driven by appetitive aggression in today’s Africa and portends to retard socio-economic development with trans-generational perpetuation. The lack of post-conflict mental health policies in nearly all African countries will be discussed and best-practice recommendations made.

## Mass Traumatization in Africa: War and Persecution

### History of Mass Trauma in Africa

Trauma, especially war-related mass trauma, is endemic and enigmatic in Africa stretching over 600 years. The 400+ year (1,451 to 1,870) history of the trans-Atlantic slave trade was associated with incessant slave raids which fueled age-old ethnic rivalries and migratory movements on the continent (4). These were then followed by 100 years of the wars of foreign colonization in what Europeans felt was their duty to “civilize” the African: the so-called “White Man’s Burden.” This was characterized by the three 3Cs of “conquer, convert, and colonize.” This period was characterized by intense foreign-imposed religious wars as competition for the African souls raged, being waged by European Christians (Catholic *vs*. Protestant) and Arab Moslems (Islam) with their African converts. This period witnessed some of the harshest and most cruel treatment of Africans by the European colonialist conquerors with frequent massacres and genocides of Africans by European powers (5, 6). Illustrative examples include in Namibia with documented evidence of the German tortures as “The First Genocide Of The 20th Century: Eurocentric Annihilation of the African Blood in Namibia 1904–1907 (7)” (6). Much inhuman treatments of humans by humans have occurred throughout history e.g., genocides of the indigenous peoples of the Americas and Australia, the Nazi Holocaust etc. However, this chapter will only deal with the negative impact of war-traumatization and persecution of populations on the African continent.

Following the period of European conquest and colonization came the wars of independence as Africans fought to liberate themselves from colonial rule. At post-independence, however, African warfare has continued in the form of today’s insurgencies, cessation movements, cross-border conflicts, fundamentalisms, and political/governance wars. In all this war-trauma the African peoples have endured stress and anxiety of unimagined proportions.

### Current War Traumatization in Africa

Research on the African continent, over the past two or so decades, has documented horrendous trauma and stressor-related mental health sequels, following the never ending enigmatic war-trauma on the African continent (3, 8). This has included reports of the common trauma syndromes of post-traumatic stress disorder, the anxieties, depression, psychosis, traumatic brain injury, epilepsies, and other physical injuries, all with their attendant complications/associations including substance abuse, epidemics e.g., HIV, cholera, Ebola etc. There have also been reports of unusual or atypical/uncommon presentations of trauma sequels in Africa such as dissociative disorders, spirit possessions (9), somatization syndromes (3), rape trauma syndrome (10), mass hysteria, or “demon attack disease” (11, 12), cult indoctrination syndromes (13), and controversial ones like nodding syndrome in Uganda (14–16). There are also new and re-emerging stress threats to the African continent posed by the ongoing mass traumatization including mass radicalizations, brainwashing, fundamentalist fanatics or religionists, destructive cults, jihadists, suicide bombers, genocidaires, appetitive aggression, child soldiers, and finally the problem of population displacements, migration, and the re-emergence of slavery (17). All this traumatization contributes to the daily mental distress felt everywhere in Africa as will be elaborated below.

### Magnitude of the Problem of Mass War-Trauma in Africa

In the past 30 years, more than three quarters of African countries have been involved in warfare in one form or another resulting in countless losses of life and causing untold misery to the common African (1, 3). Millions of Africans have been traumatized, displaced, impoverished, diseased, starved, or forced to migrate due to war. Most African countries don’t make guns, yet trade in small arms is proliferating worldwide (18). The biggest manufacturers of weapons are the rich developed countries. Many African countries have huge and unmanageable health problems (physical, mental, social, and ecological) as a consequence of prolonged militarized conflicts (3). Wars break out every year. The weapons used have become more deadly fuelled by the international trade in small arms. Developing countries have become the testing grounds of new weapons.

In today’s wars, civilian deaths far outnumber the deaths of the fighting soldiers. In World War I the ratio of Civilian : Soldier deaths was 1:10; today the ratio is 10:1 (19). In most African wars, civilians are being targeted e.g., rape of women, using civilians as human shields, child soldiers, genocides, internally displaced peoples (IDP), refugees etc. (20). Warfare has moved from battlefields to urban warfare. Targeting of civilian infrastructure is common as soft targets such as churches, mosques, markets, shopping centers, college campuses. Ideologies and fundamentalisms drive today’s wars which makes them intractable and unwinnable as we have seen in many African terrorisms. Africa has joined in the militarization of previously peaceful endeavors e.g., science, medicine, space, social media, information technology (IT), drones etc. The causes of today’s wars in Africa are difficult to discern. Many are enshrined in political and governance dilemmas, in the fight for the control of resources and territory. These form the majority of the civil wars and insurgences e.g., Nigeria, Sudan, Uganda, South Sudan, Libya, Eritrea, Ethiopia, Burkina Faso, Central African Republic etc. Others have to do with governance issues e.g., Egypt, or fundamentalist, religious, or racist connotations e.g., Somalia, Sudan, or old ethnic rivalries e.g., Rwanda, Burundi. Human rights abuses, dictatorships, political repression, and election problems account for yet another group e.g., Kenya, Democratic Republic of Congo etc. Many African leaders rule for decades and keep on changing constitutions to ensure their continued rule. Global forces, especially the control of markets and resources (oil, minerals) or international hegemony (economic, ideological, cultural, trade etc.) and fundamentalisms (religious, racism) have fuelled many proxy wars in Africa by heating up nationalisms and centuries old ethnic conflicts. Often global forces align themselves with local forces to cause what is termed as “glocal” factors that fuel conflict e.g., greed and corruption, weapons industry especially small arms trade (18). These exploitatively play on the African peoples’ poverty and ignorance to precipitate conflict and perpetuate wars. This is usually seen in the not too uncommon East *vs*. West tensions, war on terror, fundamentalisms etc. The result of all this fighting is massive loss of life. The African continent accounts for almost 88% of the world’s conflict-related deaths, with Asia and Middle East accounting for 9%, Europe 2%, and the Americas 1% as well illustrated by the Virgil Hawkin’s stealth conflicts map (21).

### The Events of Mass War-Trauma

In today’s wars, including those in Africa, civilians suffer the most (19). The types of traumas experienced can be grouped into physical, psychological, social, and ecological torture and these include the following (22):

**Physical torture**

- Beatings, kickings, gunshots, bombs, landmines
- Cuttings, tying and blindfolding, child soldiering,
- Disfigurements, burnings, forced labor,
- Sexual abuse (rape, public rape, gang rape, sexual slavery), defilements
- Executions, mass killings, ethnic killings

**Psychological torture**

- Threats, interrogations, accusations, abductions,
- Mock executions, incommunicado, detentions,
- Humiliations, witnessing, deprivations

**Social torture**

- Destruction of property and livestock,
- Fleeing, witnessing, displacements, migrations
- Community and family break-ups.

**Ecological torture**

- Destruction of infrastructures,
- Scorched earth policy,
- Uninhabitable environments—landmines, poisoned wells/rivers etc.

As an illustrative example, Musisi et al. (22) studied the traumatic events that people experienced in the Luwero Triangle during Uganda’s “bush” war from 1981 to 1986. In that war civilians were subjected to extreme trauma by the then government fighting forces. The **Table 1** summarizes the trauma frequency (22):

**Table 1:** Reports of trauma by the survivors of the 1981–1986 Bush War in Uganda—the Luwero Triangle.

Trauma event	Frequency
• Beatings, kicks, and cuts	50%
• Forced hard labor	20%
• Threats and interrogations	60%
• Relatives killed	50%
• Homes/property destroyed	40%
• Forced fleeing/displaced	60%
• Sexual torture (rape)	40%
• Tortured in home	70%
• Tortured in camp/detention	20%

Wars cause physical, psychological, social, and ecological destruction. They aim to destroy a people’s identity, culture, beliefs, language, food etc., in order to make them submit to newly imposed ones. Wars destroy society’s infrastructure and systems e.g., government, families, communities, economy, environment. Wars are coercive to dis-empower and indoctrinate. Torture is used to ensure submission. Rape of women is used as a weapon of war to change the genetic makeup. Some wars carry out genocides as was seen in Rwanda, Darfur etc. Such traumatic events go beyond normal human experiences and lead to the myriad of post-traumatic sequels we see in Africa, some of which go on for generations.

The victims of Africa’s wars are more likely to be civilians rather than the fighting forces. Indeed more than 70% of the casualties in recent African conflicts have been non-combatants. The majority of these are women, children, and the elderly (23–25). The poor and uneducated make up the bulk of the victims. The young and educated (especially men) flee into exile, robbing Africa of the social capital necessary for development. The survivors suffer significantly as described below:

***Physically:*** They suffer many diseases—surgical, gynecological, infections, neurological, neglected health, and epidemics. Healthcare infrastructure is destroyed. Health workers flee (22).

***Socially:*** Communities suffer increased poverty as production declines and education plummets as schools are destroyed. People’s cultures, societies, and communities are destroyed and many get displaced. Whole communities become marginalized creating trans-generational effects. Law and order breaks down giving way to militarism and political repression. Population displacements lead to refugees, internally displaced peoples (IDPs), asylum seekers, and running into exile of the educated and professional elites hence exacerbating the brain drain and flight of social capital (26, 27).

***Children of war:*** Children born of and in trauma are a cause of special concern. Many are orphans and vulnerable children with no adults to care for them creating the phenomenon of sibling headed households or COTOs (children on their own). Some are unwanted or unaccepted, being products of rape or incest. On rare occasions, as the war rages on, some become abandoned to grow up in the bush or forests as feral children. Children in war-torn areas are often orphaned, malnourished, and don’t go to school. They live in IDP camps; are malnourished, stunted, depressed, have epilepsy and as they grow up they gravitate to urban centers where they become street children or prostitutes (24, 25, 27). Many children are seen in IDP camps, in Africa, wandering about without adults to care for them. After the camps are disbanded many of such children go to live in towns as street children, begging and stealing for survival. Many girls became sex workers for survival. All these children become urban lumpen and become victims of drug and alcohol addiction as well as crime. NGOs and faith based organizations attempt to help e.g., Transcultural Psychosocial Organization (TPO), UNESCO, World Vision, Peter C Alderman Foundation (PCAF), etc. However, without concerted government policies for the care of these Orphans and Vulnerable Children (OVCs) who have been affected by war(s) a whole generation of African children will be lost. This has the potential to create transgenerational cross-over problems and future conflict. Most of these children suffer chronic traumatization in their tender years and develop Developmental Trauma Disorder, DTD (28).

***Child soldiers:*** UNICEF defines child soldier as any person under 18 years of age who is associated with an armed force or armed group in any capacity ranging from combatants to cooks, or laborers carrying loads (29). Child soldiers are often forcibly recruited, brutalized, and cruelly abused by armed groups. They are often forced to commit atrocities themselves, sometimes to members of their communities including their families. (24). They subsequently suffer massive mental health problems which make their treatment, community reintegration, and psychological rehabilitation difficult (24). Okello et al. (25) found the prevalence of PTSD in former Ugandan child soldiers ranging between 27 and 34.9% of the Ugandan child soldiers in rehabilitation. However, Amone-P’Olak et al. (27) reported 97–98% prevalence of post-traumatic stress symptoms, posttraumatic stress disorder (PTSS), not necessarily meeting full Diagnostic and Statistical Manual of Mental Disorders (DSM) criteria for PTSD. Other psychiatric problems were also common in these war-affected children, such as depression, various anxiety disorders, dissociation, somatic complaints, as well as behavioral problems like aggressive and disruptive behavior.

***Women survivors of war:*** Many women are sexually and physically abused in war as sexual and gender based violence (SGBV) is rampart in many African wars. Rape and gang rape, abduction, sex slavery, defilement, and forced marriage are common. Many young girls are lured by fighting men into sex in exchange for food, security, shelter, and financial assistance. For survival, they follow the men wherever they move, a phenomenon called “camp following” and many engage in survival sex (8). Even UN Peace Keeping Forces have been guilty of the sexual abuse of women in African conflict areas (30).

***Refugees and internal displacement:*** War causes people to flee in search of security and safety. It also causes poverty, hunger, and loss of hope for any meaningful livelihood. Violence and insecurity become everyday experiences. Whatever the cause, fighting, and violence cause anxiety, panic, and fear in the people who are forced to run in search of safety and security. These are the refugees, immigrants and IDPs, the bulk of who are children, women, and the elderly. The overwhelming majority of displaced people are hosted in developing countries, either as internally displaced peoples (IDPs) or as refugees in countries neighboring to the conflict zones (26). A few run to Europe and North America. However developing countries host the vast majority of refugees as internally displaced peoples. This accounts for 13.9 million refugees under the United Nations High Commissioner for Refugees (UNHCR) mandate or about 86% of the world’s refugees. Sub-Saharan Africa, as a region hosts around 30% of global refugees. Most African refugees remain within the African region where they face appalling situations (26). They often suffer traumatic events not only in their countries of origin but also in their countries of resettlement. Moreover, their bitterness, past and continued trauma, as well as the stigma and discrimination they face make these refugees and immigrants vulnerable to development of mental health problems, not only as individuals but also in families and communities wherever they have gone (3). Their mental health problems are related to various phases of the displacement experience including preflight, flight, and resettlement. The preflight phase may include, physical and emotional trauma to the individual or family, the witnessing of murder, and social upheaval. Flight involves an uncertain journey from the host country to the resettlement site and may involve arduous travel, refugee camps, and or detention centers. The resettlement process includes challenges such as the loss of culture, community, and language as well as the need to adapt to a new and foreign environment. These experiences are risk factors to mental health problems common of which are post-traumatic stress disorder (PTSD), major depression, generalized anxiety, panic attacks, adjustment disorder, somatizations, and substance abuse. Many commit suicide (31). The incidence of their diagnoses varies with different populations and their experiences. Different studies have shown rates of PTSD and major depression in settled refugees to range from 10 to 40% and 5 to 15%, respectively. Children and adolescents often have higher levels with various studies revealing rates of PTSD from 50 to 90% and major depression from 6 to 40% (24, 25, 27, 32).

With the recent increase in mass violence worldwide, has come an unprecedented upsurge in the numbers of refugees, asylum seekers, and displaced peoples, which has created the world’s immigration crisis of today. No country is spared. With this have come nationalistic, isolationist and xenophobic sentiments as well as protectionist politics. This has been especially so with the increased numbers of refugee treacherous crossing of African immigrants across the Mediterranean from North Africa (especially Libya) to Europe causing worldwide sensationalization with new drastic European approaches to dealing with the refugees from Sub-Saharan Africa and the Middle East.

The increase in wars, poverty, famine, disease, as well as political repression coupled with the globalized expectation of freedom and richness has created a dangerous narrative in today’s African youth who have been manipulated into thinking that Africa is an “unlivable continent.” Many strive to go to Europe or the Middle East “at all costs” which has resulted in massive human trafficking and domestic slavery and actual re-emergence of the tear-raising slave auctions in Africa **again** in this 21^st^ Century as was shown in one CNN newscast to all over the world (17). The core cause of all of this lies in wars on the continent and their international perpetrators working with the local Africans in the exploitation of the African resources and peoples being fueled by the world’s small arms trade. Elite Sub-Saharan Africans, who come to Europe, as exiles are often commonly portrayed as “destitute or desperate.” However, these are often relatively well educated Africans who come from moderate socio-economic backgrounds. They move because of a general lack of opportunities, fear of persecution and violence, to escape political repression or a combination of these (26). They add to the brain drain from Africa.

## Mental Health Problems of Mass Trauma in Africa

### Common Mental Disorders: Anxiety, Depression, and Post-Traumatic Stress Disorder

#### Anxiety and Depression

For years in Africa, mental health problems consequent to war were seen as annoying “anxieties and depressions” not suitable for treatment with western medicine, hence relegating them to traditional and faith healers (2, 33). Generally, the term “anxiety” is defined as distress or uneasiness of mind caused by fear of danger or misfortune; a state of apprehension and psychic tension (34). Trauma causes psychological ill-health which, before DSM 5 (35), was classified as a form of anxiety disorder called post-traumatic stress disorder (36). Today, medically, or more specifically psychiatrically, anxiety is defined as a disorder characterized by a state of excessive fear, worry, and apprehensive expectation occurring on more days than not about a number of potentially stressful events, situations, or activities such as happens in severe illness, famine, disasters, or war which often causes trauma and ill-health mentally, physically, and socially (35). Medical psychiatry also stipulates that anxiety can arise “from without.” Using their culture-specific explanatory models of illness, traumatized Africans have exhibited varying health seeking behaviors following their “anxieties.” The term “culture-bound PTSD syndromes” (37) will be discussed in the broader context of treatment, rehabilitation, and prevention of trauma-related stress and anxiety as seen today on the African continent and worldwide. War causes trauma and ill-health. The American DSM IV classified post-traumatic stress disorder (PTSD) under the category of anxiety disorders (36). However, DSM-5 (35) differentiates anxiety disorders as a distinct category from “trauma and stressor-related disorders” under which PTSD is now classified although its diagnostic criteria remain the same and still contain a significant dose of anxiety symptomatology.

This chapter argues that chronic warfare, as a lived experience has caused significant classic anxiety, depression, and other mental illnesses as well as general ill-health in many African communities and retards development (1–3). Wars, as traumatic events, have brought on a specific constellation of severe, prolonged, emotional, and physical disabling symptoms, especially war-related post-traumatic stress disorders (PTSD), depression, and anxiety with varying symptom expressions (2, 3, 33, 38). At a more general level, psychic trauma occurs when an individual is exposed to an overwhelming event that results in helplessness in the face of intolerable danger, anxiety, and instinctual arousal. In as much as stress affects everyone, severe traumatic events (as in war) tend to be overwhelming, shattering a person emotionally and leaving a feeling of total helplessness. Such as person is faced with a threat to life, a risk of injury, or a loss of security and is at a critical moment when usual coping mechanisms seem to fail (39). Hence, as argued in this chapter, the history of PTSD in Africa is closely tied to the continent’s wars and these have caused a state of continuous anxiety, depression, and psycho-social disability stretching over centuries on this continent.

#### Post-Traumatic Stress Disorder

Stress affects everyone as change and adjustment are part of life as events unfold nearly every day. However, some events can be traumatic and overwhelming, shattering a person emotionally and leaving a feeling of total helplessness. This happens when a person is faced with a threat to life, a risk of injury, or a loss of security or sanity when usual coping mechanisms fail (39). Psychic trauma occurs when an individual is exposed to an overwhelming event that results in helplessness in the face of intolerable danger, anxiety, and instinctual arousal. Such traumatic events can bring on a specific constellation of severe, prolonged, emotional, and physical disabling symptoms, an illness now known as post-traumatic stress disorder or PTSD (39).

The American DSM 5 (35) and the International ICD 10 (40) define post-traumatic stress disorder (PTSD) as consisting of a set of symptoms which make the PTSD diagnostic criteria. These can be summarized as follows:

**A**. **Experiencing, witnessing,** being confronted by or hearing about a traumatic event with threat to life, physical integrity to self or others and causing fear, helplessness, or horror.
**B**. **Re-experiencing the traumatic** event as recollections, intrusive thoughts, nightmares, or flashbacks etc.
**C. Avoidance of reminders** of the traumatic event in thoughts, situations, places, including poor recall.
**D. Hyper-arousal** characterized by poor sleep with nightmares, increased irritability, anger outbursts, hyper-vigilance, startle response, or increased autonomic activity.
**E**. **Duration of symptoms for more than 1 month** and causing significant distress to the ones affected.
**F. There can also be culturally influenced symptoms** such as dissociations, spirit possession states, and somatization.
**G. Impairment** of social and occupational/school functioning.

PTSD is often associated with complications such as depression, panic attacks, phobias, substance abuse, psychosis, and physical medical complications depending on the trauma itself e.g., traumatic brain injury, seizures, sexually transmitted diseases (STDs) including HIV, vaginal fistulae, fractures, neglected diseases, family dysfunction etc. (41). The universality of PTSD symptoms as existing in different cultures has been the subject of much research and debate. Bracken et al. (33) and Summerfeild (38) argued that post-traumatic stress disorder (PTSD) is a peculiar construct of the West and denied its universal application to nonwestern cultures. However, various workers have described a core set of symptoms found in all cultures and societies as constituting the core syndrome of PTSD as described in DSM-5 (35) and ICD-10 (40). Boehnlein (42) studied PTSD symptom expression in different cultures and the cultural interpretations of common physiological processes in PTSD e.g., nightmares. He concluded that by listening simultaneously to the literal (spoken) language, knowing cultural metaphors and observing somatic (body) language, one is led to a more comprehensive understanding of human suffering in the care of the traumatized (42). Thus various workers now agree on the varied expression of psychological distress in different cultural settings hence giving rise to the notion of “post-traumatic culture-bound syndromes” (43). The post-traumatic stress symptom constellations evolve in a timely fashion which can be interpreted as constituting sub-types of PTSD as follows:

***Adjustment disorder:*** Here the traumatic event may be a usual or common human experience, may be transient, not as severe and may be reversible or overcome-able.
***Acute Stress Disorder:*** Here, the PTSD symptoms occur within 30 days of the trauma.
***Acute PTSD***: Here the duration of PTSD symptoms is 30 to 90 days.
***Chronic PTSD:*** Here the duration of PTSD symptoms is 90 or more days.
***Delayed Onset PTSD*:** Here PTSD symptoms occur within 6 months or later of the traumatic event.

#### Complex Post-Traumatic Stress Disorder and Post-Traumatic Stress Disorder in Children: Developmental Trauma Disorder

Herman (44) described the concept of complex PTSD as a result of a severe or protracted and repeated traumatization of an individual which leads to a complex process of psychological reactions where the existence of previously held values and views of the world is doubted leading to changes in personality, beliefs, and the distrust of people (44). In children complex PTSD presents as developmental trauma disorder (DTD) and the manifest symptoms depend on the age of child, the nature of the trauma, the repetition of the trauma, the gender of the child, and support systems available to the child (28). DSM 5 (35), however tends to limit children’s response to trauma as manifesting in the form of either reactive attachment disorder (over-anxious attachment) or disinhibited social engagement disorder (or disinhibited attachment). This is limited as children show varying symptoms consequent to trauma. Children affected by war in Africa have exhibited various forms of developmental trauma disorder (14, 24, 32).

Mass trauma, as happens in war, is almost always accompanied by high rates of PTSD in both adults and children. It denigrates respect for human life, personal dignity, and what constitutes a good and meaningful life (19, 43). Communities often resort to their traditions to reconstruct their disrupted life. It is through cultural traditions that man values human life, constructs the meaning of life and respects personal dignity and that of others. In Africa, this is embodied in the concept of Ubuntu or “humaneness” (43).

Often, in Africa, unusual forms of PTSD may appear as forms of complex PTSD. These do not fit the usual PTSD diagnostic criteria, are often rare, controversial in description and presentation and may be restricted to one locality or group. Often they are associated with physical disorders and could be gender, age, location, culture, or religious specific. They are difficult to explain using the commonly stipulated PTSD disease criteria and could be interpreted as culture-bound PTSD syndromes. Researchers may write about them as isolated case reports and they often present diagnostic and management difficulties with much debate as to their diagnosis, the supposed causes and treatment approach. In Africa, these atypical PTSD syndromes may present with much drama as dissociative symptoms (psychological or somatic), spirit possession, or complex depressive symptoms with much somatization but with a history of past traumatic events. They are often treated by traditional healers by abreactive catharsis, rituals, and/or psychodrama (9).

## Atypical Mental Health Problems of Mass Trauma in Africa

Unusual forms of PTSD and other mental sequelae appear in Africa as forms of complex PTSD and mass anxieties but with strong cultural and quasi-religious influences. These do not fit the usual definition of PTSD or other known mental syndromes as classically defined. They could be rare, controversial, and restricted to one locality or group. Often they are associated with physical disorders and may be gender, age, culture, or religious specific or confined to a specific geographical location. They are difficult to explain using common DSM-5 or ICD-10 disease criteria and could be interpreted as “culture-bound PTSD syndromes” (3). Researchers write about them in isolated case reports or they get mislabeled as some other known disorders. They often present diagnostic and management difficulties with much debate as to their diagnosis, supposed causes, and treatment approaches. They will be described below.

### Dissociative Symptoms and Mass Hysteria

Van Duijl, et al. (9) reported on the presentation of PTSD symptoms as dissociative and possession states in traumatized refugee populations in Uganda. They were often accompanied by depressive symptoms and treated by traditional healers using traditional techniques of abreaction, catharsis, rituals, and psychodrama. Nakalawa et al. (12) also reported on these in post-conflict areas in Northern Uganda as well as mass hysteria, commonly referred to as “demon attack disease.” Kaggwa (11) reported on similar phenomena in East Africa following the immediate post-independent anxieties. This was a time following much traumatic anxiety and uncertainty in East Africa which accompanied the struggles for independence e.g., the MAU-MAU insurgency in Kenya (45) Rataemane et al. (46) described cases of mass hysteria in South African girl school children in the post-apartheid uncertainties. Mass hysteria was common in Europe in the middle ages e.g., the dancing manias in France (47). These mass hysterias present as spontaneous en mass development of identical symptoms among people sharing common attributes (e.g., being in same IDP camp or school or village) and who believe they have been made ill by outsiders or outside agents (witchcraft or demons etc.). They are often preceded by mass psychic trauma and are characterized by intense mass affect, anxiety, and dissociation of the manifest symptoms from consciousness. In Africa they are often attributed to demons and wronged angry spirits of the land or the dead. In today’s Africa, attempts at treatment by prayers and traditional healers have often failed. Successful treatment or intervention has been reported to be by identification of and dealing with source of conflict, isolation, and treatment of the index case and dispersal of the affected others (12). Appreciating the cultural explanatory model of the mass malady helps in understanding the phenomenon and effecting healing of the affected community.

### Cult Indoctrination Syndromes

A cult is defined as “A system of religious veneration and devotion directed towards a particular figure or object by a group of people having beliefs or practices regarded by others as strange or as imposing excessive control over members” (48). Cults often form following brainwashing and indoctrination by a charismatic (cult) leader who demands total obedience and loyalty such as what was seen in the Stockholm Syndrome. Cult leaders may lead religious or military movements. They demand obedience and total loyalty from recruits. Personal feelings are suppressed and members seem content and enthusiastic to carry out their leaders commands at all times. They adapt a drastic but total alteration of their value system, worldview and exhibit a reduction of cognitive flexibility and adaptability. They appear “anxiety-less” as they develop a narrowing, blunting and distortion of affect with apparent psychological regression. They have absolute sincerity as demanded by their leader who often demands group isolation from family and society. They often adapt a code of silence. They experience physical changes including weight loss and deterioration in physical appearance, usually from many days of forced starvation as a form of religious fasting or self-sacrifice. They may develop mask-like facies, blank stares or evasive eyes, and a puppet-like cheeriness.

Cults are seen with increasing frequency in traumatized Africa or following epidemics. Many destructive and doomsday cults have been seen on the continent. Examples include the Kibwetere’s doomsday Kanungu cult in 2000 in Uganda in which more than 1,000 people died (13). Kibwetere led the “Movement for the Restoration of the Ten Commandments of God” cult movement in Uganda in which thousands died in a deliberately set inferno set in his church with the promise “to go to heaven.” All this was in the wake of the AIDS epidemic and Uganda’s civil wars. Other cults have been seen as religious revival churches to assuage the mass anxiety, helplessness, and hopelessness in populations faced with war, poverty, famine or epidemics, and constant uncertainty about the future. Indeed many evangelistic missions and missionaries abound in today’s Africa promising riches and heaven to the suffering converts (49). Other cults have been armed and militarized and destructive in nature. They recruit followers by promising magical solutions to governance or political problems or to “purify and save their people.” Examples of these included Alice Lakwena’s Holy Spirit Movement in Uganda from which Joseph Kony’s Lord’s Resistance Army (LRA) arose (50). Alice Lakwena was a cult-priestess who recruited and mobilized an armed “holy army” which was defeated by Ugandan Government army. Joseph Kony was a catechist, a cousin of Alice Lakwena, who took over the remnants of the defeated Lakwena’s army to create an insurgency in Northern Uganda with a promise to resurrect “a pure Acholi tribe free of contamination from outsiders and then form a government based on the 10 biblical commandments” (50). Other militaristic fundamentalist cult-like armies have also been seen in Africa including Boko Haram and Al Shabab, which seem to be off-shoots of Al Qaeda (51).

Most of these groups arise because of the ever-present anxiety we see in Africa, most of which stems from war, poverty, famine, and mass disease or epidemics as well as abuse of Human Rights; all mixed with ignorance and the African traditional beliefs in magic and supernatural forces. They cause much discomfort among Africans who get manipulated into believing them to be “saviors” as a means to escape the mass poverty, suffering and misery, and the attendant constant anxiety, all of which stem from chronic warfare.

### Rape Trauma Syndrome

This was first described and reported by Burgess and Holmstrom (10) as occurring in women in the Democratic Republic of Congo, DRC, who had suffered repeated gang rapes and sexual slavery. Rape of women and young girls, including gang rape, was very commonly reported in DRC (41, 52) and was also seen in northern Uganda and other areas of conflict in Africa (8, 23). Rape is not usually reported by the women victims for cultural fears of shame and rejection by their husbands or reprisals by their rapists. Rape trauma syndrome usually presents as associated to other psychiatric disorders including chronic (lower) abdominal pain, depression, amnesia, dissociation, conversion disorder (lower limbs weakness), or suicide attempt (53). The women may present after many years of failed treatments including gynecological referrals and treatments for sexually transmitted infections including HIV/AIDS. Treatment of the underlying complex PTSD using antidepressants, anxiolytics, anti-arousals (prazosin), and culturally sensitive trauma-focused cognitive behavioral therapy CBT is useful.

### Nodding Syndrome and Starvation Syndromes

Nodding syndrome was reported in war-afflicted Northern Uganda and South Sudan (54). It presents as a chronic debilitating illness affecting children aged 3–18 years. Winkler (55) had reported on sporadic cases of nodding disease in Tanzania, which were not related to trauma and had classified them as atypical epilepsy. In Northern Uganda’s war conflict-affected areas, reports of nodding syndrome first appeared in 1997 (54). Nodding syndrome, NS, was characterized by malnutrition, stunted growth, mental retardation, and seizures in about 50% of the cases leading some researchers to designate it as a neurological epilepsy disorder (15, 16). However, the affected Acholi people called it *“luc luc* **or** *yengo wic”* and recognized it as a new disease in their midst and related to the LRA insurgency war. They did not call it epilepsy i.e., *“jake* **or** *two oderu,”* these being more familiar terms to mean epileptic fits which they had always seen. They believed nodding syndrome to be caused by “cen” or dead peoples’ spirits which had come to haunt the living for a variety of reasons including wrongful deaths and improper burials. Reports of nodding syndrome first appeared in Northern Uganda in 1997 and reached epidemic proportions in 2000-2003 when people were moved into IDP camps (14). Investigations for infectious (onchocerciasis) and toxic (rotting foods, chemicals from ammunitions etc.) were inconclusive as to cause, treatment, or outcome. Psychiatric studies of clinical evaluations and field observations revealed that nodding syndrome children had been exposed to severe war-related psychological and physical trauma as well as non-specific CNS insults including untreated CNS infections (malaria, meningitis) and malnutrition (avitaminosis) possibly causing seizures. Many children suffered post-traumatic stress disorder (PTSD) or developmental trauma disorder (DTD) and depression. No more **new** cases of nodding syndrome appeared after the LRA war ended and the IDP camps were disbanded in 2007 (14, 16). However, the nodding syndrome children who had brain-damage from CNS insults continued to suffer and exhibit features of mental retardation and other neurological developmental anomalies. Family therapy using group interpersonal psychotherapy (IPT-G) helped families of NS affected children by lowering their depression and anxiety to cope better and look after their NS children (16). No other measures effectively treated NS except for symptomatic treatment of any accompanying seizures or depression and malnutrition (14). Those NS children who had no neurological problems did well and have gone on to lead more or less normal lives after the war.

### Appetitive Aggression

Generally, people with PTSD following trauma, face many challenges in their lives including both enacted and internalized (self) stigma, the latter being most harmful (56). Internalized or self-stigma is defined as “a state in which an individual accepts and agrees with societal prejudices about their particular condition and applies this to oneself (56). It is associated with lowered self-esteem and hope and may carry on for generations and negatively influences behavior. It perpetuates symptoms of PTSD, depression, anxiety, substance abuse, as well as aggression leading to violence in communities as “appetitive aggression.” Appetitive aggression is defined as the “perpetration of violence and/or the infliction of harm to another person for the purpose of lessening one’s pain, for relief of anxiety/inner tension or just for “fun.” Traumatic experiences lead to emotional deregulation which in turn leads to easy irritability and ultimately to aggression (e.g., startle response, hyper-vigilance etc.). The resultant violence results from a form of “reactive aggression” and the need to revenge for relief of the inner tension/anxiety, hence creating a pattern of appetitive aggression (57). Individuals who are exposed to earlier trauma often perpetrate violence or inflict pain/harm to others (victims) for purposes of experiencing violence-related enjoyment although this diminishes with higher levels of violence. Appetitive aggression temporarily reduces risk of trauma-related distress and is hence adaptive for survival in a violent environment (56). Sometimes, there are powerful social and psychological rewards gained from appetitive aggression, especially from people in authority. This reduces their vulnerability to the trauma-related psychological distress hence the seeming protective effect and tendency to self-propagate (57). Appetitive aggression could partly explain the increasing types of violence reported in post-conflict communities including domestic violence, gender based violence, intimate partner violence, child abuse, mob/vigilante violence, and the vicious cycles of political repressions and torture including “riot suppression.” “Toxic stress” as is often seen in the former child soldiers and returned girl abductees drives appetitive aggression. Their appetitive aggression is an adaptation to their earlier experiences of violence as a form of PTSD with startle responses as they feel vulnerable, have low self-esteem and feel easily threatened. They develop internalized stigma (self-stigma) and are always suspicious of others’ intentions (paranoid). They thus “attack pre-emptively.” Examples of this are returned child soldiers who find it difficult to sit in classrooms and obey the authority of teachers to benefit from the learning obtained from formal school education. These often feel out-casted and have low self-esteem and feel self-stigma. Returned formerly abducted girls who suffered trauma and sexual abuse also felt the same way. Treatment provided in the form of narrative exposure therapy (NET) could help them in reconstructing an autobiography of themselves and a memory of their past experiences (56). This provides them an understanding of why they feel and behave the way they do (appetitive aggression), reduces their inner fears and tensions, and may thus reduce their violent or avoidance tendencies. This has not been possible to do in Africa on a wider scale due to limitations of the needed knowledge and expertise to carry out NET on a mass scale in former combatants of Africa’s wars. Researchers have argued that many of today’s African political leadership are former combatants who have PTSD and may be unconsciously caught up in vicious wars of appetitive aggression. Appetitive aggression has the potential for the trans-generational transmission of trauma and may be the psychological force driving genocidaires.

## Policy, Conclusions, and Recommendations

### Policy

Historically, for over 600 years, Africa has been the scene of significant war-related mental distress and uncertainties; from ethnic rivalries fomenting war-raids, wars of slavery, European colonization of African peoples (to conquer, convert, and colonize), to wars for independence and now the post-independence wars fomented by fundamentalisms, competing political ideologies/persecution, racism, and the struggle for control of resources and lastly population movements and human trafficking. All of these have been driven by warring. Over the years, this has shaped many African peoples’ attitudes, politics, and health seeking behaviors. Today African peoples are anxious over a number of issues including sexuality, gender, health, disease, poverty, modernity, climate, politics, culture, religion, ethnicities, race, economies, governances, war, displacement, immigration, and fears that they may, once again, lose control of their continent.

From a policy perspective, mass trauma is a global health problem whose causes, perpetuates, solutions and preventions elude nations. Despite such chronic massive traumatization on the African continent, with the consequent mental health disability, there is virtual absence of national mental health policies in African countries, let alone national post-conflict mental health policies (1, 3, 20, 58–60). The African Union Policy on Post-Conflict Reconstruction and Development (PCRD) did not mention mental health in its recommendations (20). In studies of mental health policy in African countries, Omar et al. (61) and Flisher et al. (58) pointed to the lack of or very inadequate mental health planning in African countries. Many researchers point to the non-prioritization of mental health issues and the lack of mental health awareness as underlying the absence of mental health policies, funding, and services in post-conflict African countries (1, 3, 59). Sankoh et al. (60) decried the lack of mental health research and publications in Africa. Of recent some research studies and publications on post-conflict mental health have appeared in the literature (29, 62–64) with but one journal on the continent being dedicated to mental health trauma in Africa: the African Journal of Traumatic Stress now being underwritten by Health Rights International (www.petercaldermanfoundation.org/ajts) (65). The policy recommendations have mainly followed the WHO-recommended mental health interventions in low-resource settings—mainly the WHO Mental health gap action program and (mhGAP) (66), Psychological First Aid (PFA) (67) guidelines (WHO, Geneva). The following is but an outline of policy recommendations regarding the least that needs to be done (37):

1. Psychosocial Needs Assessment: To start by doing scientifically based local research to inform policy makers of the need to have post-conflict mental health recovery programs. This should address the need to treat current victims, reconstruct affected communities, conflict resolution, peace building reparations, and redress. The latter is important as reparations without justice tend to perpetuate future conflict.
2. These intervention programs must be nationally organized based on policy legislation and financing, multi-disciplinary approaches, local monitoring, and control of international collaborating agencies.
3. The mental health intervention policy should be linked to socio-economic development and human rights.
4. The treatments must be based in primary health care settings to avoid stigmatizing victims. There has to be local input and participation in the provided interventions which must be culture and situation sensitive.
5. All African countries should endeavor to provide physical and mental healthcare for internally displaced peoples, immigrations, and refugees.

### Conclusions and Recommendations

There has been many wars, persecutions, and traumatization the world over. However, by emphasis, this chapter only dealt with African communities. From a medical point of view, we see many diseases and behaviorisms as a consequence of the chronic warfare and persecution in Africa. This chapter has shown how the trauma, as seen in African wars, continues to cause much mental distress and morbidity in many African communities and to retard socio-economic development. Diagnoses such as post-traumatic stress disorder, depression, and anxiety are increasingly seen today’s Africa with their various presentations. Atypical forms of these mental problems often manifest given the varying explanatory models of illness causation in the different cultures in Africa. In their health seeking, Africans have employed various methods to cope with or reduce their mental ill-health including traditional healings, faith healings, cleansing rituals, reconciliations, and conflict resolutions, but the trauma still goes on. Africa needs to re-think and curb warring and its deleterious ramifications including re-insurgent slavery.

The question whose answer eludes many in Africa is social justice for all. How will Africa forge a path to peaceful development, freedom, human rights observance, and good governance? Many decry the long legacy of colonialism which divided up African peoples with countries sketched out by European powers and the latter who continue to control Africa’s resources. There are no easy answers and certainly no simple specificities/specifications especially in today’s competing global forces in diverse African cultural settings. The following are suggested recommendations:

1. Universal education for all including all ethnicities, genders, social classes, religious groupings, and political persuasions which must include human rights education.
2. A political will by African leaders to entrench participatory democratic governance in their countries and keep way from perpetual dictatorships, wars, and persecutions.
3. A deliberate education that concietizes the people of Africa to know the historical and socio-economic origins of their problems and to understand the global forces perpetuating their present socioeconomic problems including the need to control African resources and affairs by Africans and for Africans irrespective of race, religion, ethnicity, gender, creed, or political/ideological persuasion.
4. Entrench, through school and community education, a universal respect for human rights; and to avoid war and promote peace building and conflict resolution.
5. All African countries should be signatories to the United Nations Convention Against Torture (UNCAT) (68) as they are all signatories to the UN Human Rights Council Resolutions (69). They should ratify UNCAT and put it in their domestic laws.
6. African countries should sign up and support the International Criminal Court (ICC) (70) and they should endeavor to have a respectable functioning African Court on Human and Peoples Rights (71) with ready accessibility to the ordinary African.

## Data Availability Statement

The datasets generated for this study are available on request to the corresponding author.

## Author Contributions

The authors carried out primary research, reviewed literature, treated subjects, collected data, analysed data and prepared the manuscript.

## Conflict of Interest

The authors declare that the research was conducted in the absence of any commercial or financial relationships that could be construed as a potential conflict of interest.

The handling editor declared a past co-authorship with one of the authors SM.

## References

[1] Musisi S Mass trauma and mental health in Africa. Afr Health Sci J (2004) 4(2). PMC2141620 15477185

[2] Pringle Y (2019). Psychiatry and decolonization in Uganda: mental health in historical perspectives. Ch 7, 177–207. 10.1057/978-1-137-60095-0_7

[3] Musisi S War and mental health in Africa. In: Njenga F Acuda W Patel V Maj M, editors. Essentials Of Clinical Psychiatry For Sub-Saharan Africa. Milano, Italy: Masson Publications (2005).

[4] Reader J Africa, A biography of the continent. In: Penguin Books. London, UK: Vintage Publishers. (1998).

[5] Sarkin J Germany’s Genocide of the Herero: Kaiser Wilhelm II, His General, His Settlers, His Soldiers. Cape Town, South Africa: UCT Press (2010).

[6] Olusoga D Erichsen CW (2010). The Kaiser’s Holocaust: Germany’s Forgotten genocide and the colonial roots of nazism. Faber Publishers, https://www.goodreads.com/book/show/8250985-the-kaiser-s-holocaust.

[7] Olusoga D Erichsen C The Kaiser’s Holocaust: Germany’s forgotten genocide and the colonial roots of Nazism. (Philadelphia, USA: Faber & Faber Available at https://www.google.ca/search?q=german+namibia+genocide.

[8] Kinyanda E Musisi S War traumatisation and its psychological consequences in women of Gulu district. In: Violence Against Women and Children: A Review of women’s studies, VOL XI Nos 1&2. University Center of Women’s Studies: University of Philippines Press (2002).

[9] Van Duijl M Nijenhuis E Komproe IH Gemaat HB de Jong JT Dissociative symptoms and reported trauma among patients with spirit possession and matched controls in Uganda. Culture Med And Psychiatry (2010) 34(2):380–400. 10.1007/s11013-010-9171-1 PMC2878595 20401630

[10] Burgess AW Holmstrom LL Rape trauma syndrome. Am J Of Psychiatry (1974) 131(9):981–6. 10.1176/ajp.131.9.981 4415470

[11] Kaggwa BH The problem of mass hysteria in east africa. East Afr Med J (1964) 41(12). 14252184

[12] Nakalawa L Musisi S Kinyanda E Okello ES Demon attack disease: a case of mass hysteria after mass trauma in a primary school in Uganda. Afr J Of Traumatic Stress (2010) 1(1):43–8.

[13] Kanungu Tragedy (2000). Kibwetere, Uganda’s most wanted man of the Movement for the Restoration of the Ten Commandments of God cult. New Vision Newspaper Report, https://www.newvision.co.ug/new_vision/news/1300274/kibwetere-uganda.

[14] Musisi S Akena D Nakimuli-Mpungu E Abbo C Okello J Neuropsychiatric perspectives on nodding syndrome in Northern Uganda: a case series study and review of the literature. Afr Health Sci (2013) 13(2):205–18. 10.4314/ahs.v13i2.3 PMC3824492 24235916

[15] Idro R Namusoke H Abbo C Mutamba BB Kakooza-Mwesiga A Opoka RO Patients with nodding syndrome in Uganda improve with symptomatic treatment: a cross-sectional study. BMJ Open (2014) 4(11):e006476. 10.1136/bmjopen-2014-006476 PMC4244396 25398677

[16] Mutamba BB Kane JC de Jong JTVM Okello J Musisi S Kohrt BA Psychological treatments delivered by lay community health workers in low resource government health systems: effectiveness of group interpersonal psychotherapy for caregivers of children affected by nodding syndrome In Uganda. Psychol Med (2018) 48(15):2573–83. 10.1017/S0033291718000193 PMC6093795 29444721

[17] Elbagir N (2017). CNN documented Human Slave Auctions. https://www.poynter.org/news/how-cnn-documented-human-slave-auctions.

[18] Tete A (2015). Human cost of illicit flow of small arms, and light weapons permanent observer of the African Union to the United Nations. https://www.un.org/press/en/2015/sc11889.doc.htm.

[19] Mollica RF Invisible wounds. Sci Am (2000) 282:54–7. 10.1038/scientificamerican0600-54 10862423

[20] African Union, AU (2006). Policy on post-coflict reconstruction and development, PCRD. ConAict, management division. peace and security department. Commission of the African union, Addis Ababa, Ethiopia.

[21] Hawkins V Stealth Conflicts: How the World’s Worst Violence is Ignored. (Aldershot: hgate) (2008). 10.1177/02673231100250020507

[22] Musisi S Kinyanda E Liebling H Mayengo-Kiziri (2000). Post traumatic torture disorders in Uganda. A 3-year retrospective study of case records at a specialized torture treatment center, Kampala, Uganda. Torture Vol. 10 No. 3.

[23] Kinyanda E Musisi S Biryabarema C Ezati I Oboke H Ojiambo-Ochieng R War related sexual violence and it’s medical and psychological consequences as seen in Kitgum, Northern Uganda: a cross-sectional study. BMC Int Health Hum Rights. (2010) 10:28. 10.1186/1472-698X-10-28 21067571 PMC2996351

[24] Betancourt TS Speelman L Onyango G Bolton P Psychosocial problems of war-affected youth in Northern Uganda. Trancultural Psychiatry (2009) 46(2):238–56. 10.1177/1363461509105815 PMC2775515 19541749

[25] Okello J Onen TS Musisi S Psychiatric disorders among war-abducted and non-abducted adolescents in Gulu District, Uganda: a comparative study. Afr J Psychiatry (2007) 10:225–31. 10.4314/ajpsy.v10i4.30260 19588031

[26] Kasozi J (2017). The refugee crisis and the situation in Sub-Saharan Africa. http://oegfe.at/2017/06/the-refugee-crisis-and-the-situation-in-sub-saharan-africa/.

[27] Amone-P’Olak K Mental states of adolescents exposed to war in Northern Uganda: finding the appropriate methods of rehabilitation (1). Torture (2006) 16(2):93–107. 17251641

[28] Van Der Kolk BA Developmental Trauma Disorder: Towards A Rational Diagnosis For Children With Complex Trauma Histories. APA PsycNET. Psychiatric Annals (2005). 35(5): 401–408

[29] UNICEF. Child Soldiers In Africa - The Paris Principles (UNICEF 2007): Principles and guidelines on children associated with armed forces or armed groups. Med War (2007). https://www.unicef.org/mali/media/1561/file/paris prnciples.pdf

[30] Lalbahadur A (2017). https://www.africaportal.org/features/peacekeeping-and-sexual-assault-persistent-stain-united-nations-image/ Peacekeeping and sexual abuse – A persistent stain on the United Nation’s Image.

[31] Kiwuwa P Odeng M (2017). Fourty eight Ugandans commit suicide in middle east. New vision newspaper, Vol 032, No. 207. Oct 18th 2017.

[32] Okello J (2014). War trauma, attachment and risky behaviour in adolescents in northern Uganda. PhD Thesis. Faculteit Psychologie en Pedagogische Wetenschappen, Universitet Gent, Belgium.

[33] Bracken PJ Giller JE Summerfeild D Psychological responses to war and atrocity: the limitations of current concepts. Soc Sci Medicine. (1995) 40(8):1995. 10.1016/0277-9536(94)00181-R 7597460

[34] Webster’s Universal College Dictionary (1997). Random House, New York, USA. Pp36. ISBN 0-517-18361-7.

[35] DSM 5 Diagnostic And Statistical Manual Of Mental Disorders. 5th Ed Arlington, VA, USA: American Pyychiatric Association, APA (2013).

[36] DSM- IV Diagnostic And Statistical Manual Of Mental Disorders. 4th Ed Washington DC, USA: American Pyychiatric Association, APA (1994).

[37] Musisi S Mollica R Weiss M Supporting refugees and victims of war. In: Hosman C Jane-Llopis E Saxena S, editors. Prevention of Mental Disorders – Effective Interventions and Policy Options. Geneva, Swizerland: WHO (2005).

[38] Summerfeild D The invention of post-traumatic stress disorder and the social usefulness of a psychiatric category. Br Med J (2001) 322:95–8. 10.1136/bmj.322.7278.95 PMC1119389 11154627

[39] Walugembe JE A pioneer look at post-traumatic stress disorder in Uganda. Afr J Traumatic Stress (2009) 1(1). (Post-humus).

[40] ICD-10 (1994). International classification of diseases, - Mental and behavioral disorders (F40-F48). World Health Organisation, Version 10, Geneva, Switzerland.

[41] Zihindula G Consequences of Gender Based Violence on Women’s Reproductive Health: HIV/AIDS and Unwanted pregnancies as immediate consequences. Germany: Lambert Academic Publishing (2011).

[42] Boehnlein JK cultural interpretations of psychological processes in post-traumatic stress disorder and panic disorder. Trans-Cultural Psychiatry (2001) 38(4):461–7. 10.1177/136346150103800403

[43] Musisi S Okello ES Abbo C Culture and traditional healing in conflict/post-conflict societies. Afr J Of Traumatic Stress (2010) 1, 2:p80–85.

[44] Herman JL Complex post-traumatic stress disorder - A syndrome in survivors of prolonged and repeated trauma. J Traumatic Stress (1992) 5(3):377–91. 10.1002/jts.2490050305

[45] MAU-MAU insurgency in Kenya (1952-1960) https://www.blackpast.org/global-african-history/mau-mau-1952-1960/.

[46] Rataemane ST Rataemane LUZ Mohlahle J Mass hysteria among learners at Mangaung schools in Bloemfontein, South Africa. Int J Psychosocial Rehabil (2002) 6:61–7.

[47] Waller J How distress and pious fear have led to bizarre outbreaks across the ages; looking back: dancing plagues and mass hysteria. Psychol (2009) 22(7):644–7.

[48] Cult definition: a system of religious veneration and devotion directed towards a particular figure or object by a group of people having beliefs or practices regarded by others as strange or as imposing excessive control over members. (2008) https://en.oxforddictionaries.com/definition/cult and http://www.dictionary.com/browse/cult%20definition?s=t.

[49] Williams RR (2013). God Loves Uganda. www.imdb.com/title/tt1874513.

[50] Gersony R The Anguish of Northern Uganda. In: . Results of a Field-based Assessment of the Civil Conflict in Northern Uganda. Uganda (1997). USAID Mission.

[51] Sheikh TL Mohammed A Nuhu FT Akande Y Coordinating psycho-social interventions for the internally displaced persons (IDPs) following insurgency in North Eastern Nigeria. Afr J Traumatic Stress (2016) 5(1):17–22.

[52] Wakabi W Sexual violence increasing in the democratic republic of Congo. Lancet (2008) 372(9655):2011–:2012. 10.1016/S0140-6736(08)61854-1 19090027

[53] Sebit MB A case study of post-traumatic stress disorder associated with conversion disorder and pain disorder in Tanzania. Afr J Traumatic Stress (2013) 3(1):33–8.

[54] WHO (2011). Uganda ministry of health. A report on the burden and epidemiology of nodding disease in the districts of Kitgum, Lamwo and Pader in Northern Uganda – August 2010.

[55] Winkler A The head nodding syndrome clinical classification and possible causes. Epilepsia. (2011) 49(12):2008–15. 10.1111/j.1528-1167.2008.01671.x 18503562

[56] Sommer J Hinsberger M Elbert T Holtzhausen L Kaminer D Seedat S The interplay between trauma, substance abuse and appetitive aggression and its relation to criminal activity among high-risk males in South Africa. Addictive Behav (2016) 64:2017) 29–34. 10.1016/j.addbeh.2016.08.008 PMC5102240 27540760

[57] Weierstall R Haer R Banholzer L Elbert T Becoming cruel: Appetitive aggression released by detrimental socialisation in former Congolese soldiers. Int J Behav Dev (2013) 37(6):505–13. 10.1177/0165025413499126

[58] Flisher AJ Lund C Funk M Mental health policy development and implementation in four African countries. J Health Psychol (2007). 12(3): 515–516 10.1177/1359105307076237 17440000

[59] Jenkins R Baingana F Ahmad R McDaid D Atun R International and national policy challenges in mental health. Ment Health In Family Medicine. (2011) 8(2):101–14. PMC3178192 22654973

[60] Sankoh O Sevalie S Weston M Mental Health In Africa. Comment. Lancet Global Health (2018) 6(9):PE954–E955. 10.1016/S2214-109X(18)30303-6 30103990

[61] Omar MA Green AT Bird PK Mirzoev T Flisher AJ Kigozi F Mental health policy process: a comparative study of Ghana, South Africa, Uganda And Zambia. Int J Of Ment Health Syst (2010) 4:24. 10.1186/1752-4458-4-24 20678205 PMC2923107

[62] Musisi S Muron J Nakku J Using Mobile Mental Health Clinics to Increase Access to Mental Health Care in Post-Conflict Rural Uganda. USA: Symposium Proceeds at the APA-San Francisco (2019).

[63] Okello J Derluyn,I Musisi,S Broekaert E The Fit Between Mental Health Needs And Programming Responses For War Affected Children In Northern Uganda. In: Derluyn I Mels C Parmentier S Vandenhole W, editors. Remember. Rehabilitation, Reintegration And Reconciliation Of War-Affected Children, Volume 11 Of The Series On Transitional Justice. Cambridge/Antwerp/Portland, Intersentia: Sofie Vindevogel, Universiteit Gent. (2012). p. 568.

[64] Nakimuli-Mpungu E Kizito W Okello J Alderman S Odokonyero R Mojtabai Group support psychotherapy for depression treatment in people with HIV/AIDS in Northern Uganda: a single-centre randomized controlled trial. Lancet (2015). 2(5): p200–7. 10.1016/j.jad.2014.03.042 26423001

[65] HealthRight International. Peter C Alderman Program For Global Mental Health. https://healthright.org.

[66] WHO Mental Health Gap Action Programme (mhGAP): World Health Organization https://www.who.int/mental_health/mhgap/en/The. 38117918

[67] WHO Psychological first aid (PFA) - World Health Organization. https://apps.who.int/iris/bitstream/10665/102380/1/9789241548618_.

[68] United Nations Convention Against Torture (UNCAT) A Summary of the Convention against Torture - Redress https://redress.org/wp-content/uploads/2018/10/REDRESS-Summary-of-UNCAT-2018.pdf.

[69] United Nations Human Rights Council’s Resolutions https://www.un.org/unispal/human-rights-council-resolutions/. Jan 3, 2019.

[70] International Criminal Court (ICC)-Hauge. https://www.icc-cpi.int/.

[71] African Court on Human And Peoples’ Rights Mwalimu Julius Nyerere Conservation Center, Dodoma Road, P.O. Box 6274, Arusha Tanzania. http://www.african-court.org/en/.

